# Elucidating Genetic Diversity in Apricot (*Prunus armeniaca* L.) Cultivated in the North-Western Himalayan Provinces of India Using SSR Markers

**DOI:** 10.3390/plants10122668

**Published:** 2021-12-04

**Authors:** Zahid Nabi Sheikh, Vikas Sharma, Rafiq Ahmad Shah, Shilpa Raina, Maha Aljabri, Javid Iqbal Mir, Naser AlKenani, Khalid Rehman Hakeem

**Affiliations:** 1 Division of Biochemistry, Sher-e-Kashmir University of Agricultural Sciences and Technology, Jammu 180009, J&K, India; sheikhzahid375@gmail.com (Z.N.S.); shilparaina70@gmail.com (S.R.); 2 Ambri Apple Research Center, Sher-e-Kashmir University of Agricultural Sciences and Technology, Kashmir 190025, J&K, India; rafiq_masoodi@rediffmail.com; 3 Department of Biology, Faculty of Applied Science, Umm Al-Qura University, Makkah 21421, Saudi Arabia; Myjabri@uqu.edu.sa; 4 Research Laboratories Centre, Faculty of Applied Science, Umm Al-Qura University, Makkah 21421, Saudi Arabia; 5 Indian Council of Agricultural and Research Central Institute of Temperate Horticulture, Old Airport Road, Rangreth, Srinagar 190007, J&K, India; javidiqbal1234@gmail.com; 6 Department of Biological Sciences, Faculty of Science, King Abdulaziz University, Jeddah 21589, Saudi Arabia; nkenani@gmail.com; 7 Princess Dr. NajlaBint Saud Al-Saud Center for Excellence Research in Biotechnology, King Abdulaziz University, Jeddah 21589, Saudi Arabia

**Keywords:** diversity, apricot, polymorphism, genotype, structure, population

## Abstract

Apricot (*Prunus armeniaca* L.) is an important temperate fruit crop worldwide. The availability of wild apricot germplasm and its characterization through genomic studies can guide us towards its conservation, increasing productivity and nutritional composition. Therefore, in this study, we carried out the genomic characterization of 50 phenotypically variable accessions by using SSR markers in the erstwhile States of Jammu and Kashmir to reveal genetic variability among accessions and their genetic associations. The genetic parameter results revealed that the number of alleles per locus (Na) ranged from 1 to 6 with a mean Na value of 3.89 and the mean effective number of alleles (Ne) per locus 1.882 with a range of 1.22 to 2. Similarly, the polymorphic information content (PIC) values ranged from 0.464 to 0.104. The observed heterozygosity (Ho) (0.547) was found to have higher than expected heterozygosity (He) (0.453) with average heterozygosity of 0.4483. The dendrogram clustered genotypes into three main clades based on their pedigree. The population structure revealed IV sub-populations with all admixtures except the III sub-population, which was mainly formed of exotic cultivars. The average expected heterozygosity (He) and population differentiation within four sub-populations was 1.78 and 0.04, respectively, and explained 95.0% of the total genetic variance in the population. The results revealed that the SSR marker studies could easily decrypt the genetic variability present within the germplasm, which may form the base for the establishment of good gene banks by reducing redundancy of germplasm, selection of parents for any breeding program.

## 1. Introduction

Apricot (*Prunus armeniaca* L) is one of the influential fruits of the Rosaceae family that is mostly produced in temperate climates. Apricots have been divided into seven eco-geographical groups based on their origin [1]. Among all groups, the Central Asian cultivars are the most diverse and the oldest of all. These cultivars form two main gene pools such as Central Asia and Eastern Asia [2] and are characterized by high chilling requirements. Major regions for the cultivation of apricot accessions are Central Asia and China, from Kashmir to Tien-shan [3]. The Central Asian geographical region shows the richest variability [4]. Regions such as Turkey, Italy, Spain, the USA and France are widely known for producing apricot in colossal amounts [5]. In Asia, apricot is majorly produced in North-Western Himalayan regions, where it has been reported to grow wild in desert areas of Tibet that mostly remain cold, Southern regions of China and in some Northern parts of India, which include the temperate areas of Himachal Pradesh, Jammu and Kashmir and Uttarakhand that fall in the elevation range of 2000 to 2500 m above sea level. Jammu and Kashmir has rich genetic diversity of apricot, possessing both indigenous accessions cultivated through seeds and some exotic collections propagated by grafting traditional local cultivars. The exotic apricot cultivars such as ‘Harcot’, ‘Hartlay’, and ‘New-Castle’ have been introduced in different temperate areas including Jammu and Kashmir because of their highly productive nature, self-compatibility, resistance to diseases and long shelf life [1,6]. Cultivation of only a few of these commercial cultivars having commercial importance in place of diverse indigenous local cultivars may lead to genetic erosion because of a decrease in genetic diversity [7]. However, the presence of diverse plant genetic diversity as found in Jammu and Kashmir is important for increasing crop productivity and development of new cultivars. Therefore, evaluating these potential plant genetic resources is essential for future plant breeding and maintaining natural populations as viable evolutionary units in genetic resource management [8,9]. In addition, such studies help to determine the extent of genetic deviation among and within populations and reveal the processes that support these variations. Different scientists have used different morphological characters to assess the degree of diversity. However, the diversity assessment through morphological characterization is expensive, lengthy and is influenced by the environment. Therefore the DNA markers are used for plant diversity evaluation [10,11]. Various primers were significantly used in apricot to explore diversity such as amplified fragment length polymorphism (AFLP), restriction fragment length polymorphisms (RFLP) and randomly amplified polymorphic DNAs (RAPD) and ISSR [12,13,14,15,16,17,18]. Repeated DNA sequences or microsatellites are the markers that are intermittently used for diversity studies because of their even distribution throughout the genome, codominance and highly polymorphic nature [19,20]. Earlier investigations have acknowledged the considerable extent of molecular variation in *Prunus* genotypes [21,22,23,24] through microsatellites [25,26,27,28,29,30] utilized specific marker pairs devised primarily for other *Prunus* species and stone fruits [31,32,33,34,35,36]. The numbers of primers have been fabricated recently by utilizing sequence information of the apricot genome [37,38]. For diversity analysis, SSR markers have been adapted in Turkey, China, Morocco and other diverse eco-geography groups [2,3,11,39,40,41,42,43]. These studies have not only helped us to understand the molecular genetic variability and population structure of the local population but have helped researchers to advance biological research and the development of future breeding programs in *Prunus armeniaca* L. In this part of India, no such type of study has been carried out so far to evaluate the genetic diversity of the *Prunus armeniaca* L from the whole temperate areas of Jammu and Kashmir through SSR markers. Therefore, this study was undertaken to examine the genetic diversity and population structure between 50 apricot genotypes (local and exotic cultivars) and to evaluate the degree of variation among and within eco-geographical groups and subgroups of apricot germplasm taken into consideration.

## 2. Results

### 2.1. SSR Genotyping and Genetic Diversity Analysis

Genetic diversity was assessed among 50 apricot genotypes by using 46 SSR markers. The genetic parameters are shown in Table 1.

Using 46 SSR markers on 50 apricot genotypes, a total of 179 alleles were detected, and the number of alleles (Na) ranged from 1 to 6, with an average value of 3.89. Among the 46 markers, the highest number of alleles, 6 per locus, was realized with 14 markers, and the highest number of effective alleles (Ne) was observed, 2 per locus with five markers such as RPPG5-030, RPPG6-033, PacA10, PacA22 and PacA26. The PIC value varied in range from 0.104 to 0.464, with an average value of 0.320. Furthermore, the number of effective alleles (Ne) ranged from 1.1563 to 2 with an average value of 1.8821. The average observed homozygosity (Ho) was 0.4774, and varied from 0 to 1, whereas the average expected homozygosity was 0.5470 which ranged from 0.4947 to 0.8634. Similarly, the observed heterozygosity ranged from 0 to 1 with an average value of 0.5226, and expected heterozygosity (He) ranged from 0.1366 to 0.5053 and produced an average value of 0.4530. The overall average heterozygosity was 0.4483 and ranged from 0.1352 to 0.5. The Shannon’s diversity index (I) ranged from 0.2611 to 0.6931 with an average value of 0.6371, and the genetic differentiation (Fst) ranged from 0 to 0.08 with an average value of 0.0228. Different parameters showed a lot of variabilities indicating high genetic diversity.

### 2.2. Cluster and PCoA Analysis

**Figure 1:**
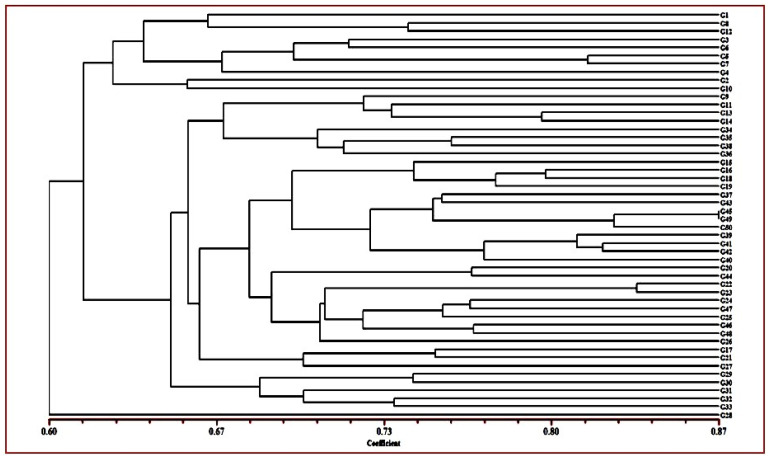
UPGMA dendrogram showing clustering of 50 Apricot genotypes based on Jaccard’s Similarity Coefficient.

The Jaccard’s similarity coefficients between the germplasms were calculated for UPGMA clustering. The diverse group of 50 germplasms were divided into three primary groups based on their genetic similarity at a distance of 0.614 as cluster I, cluster II and cluster III (Figure 1).

**Figure 2:**
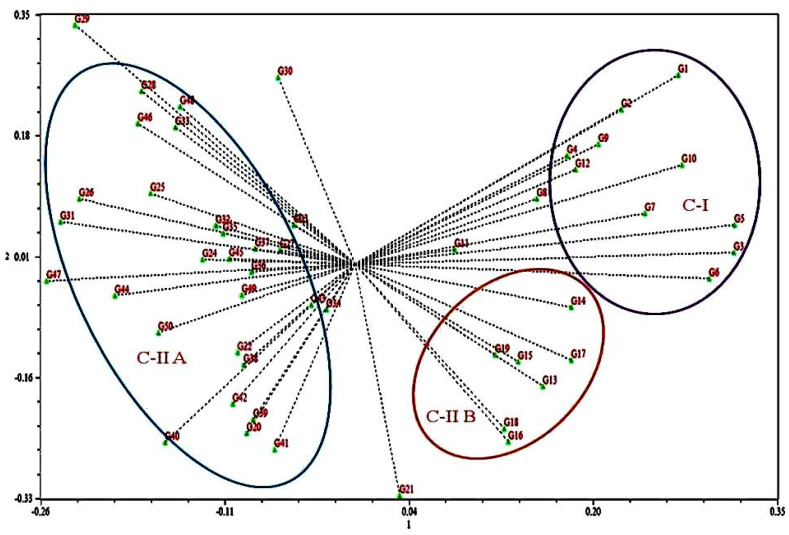
A biplot of the first two principal components of 50 apricot genotypes using 46 microsat−ellite markers.

**Figure 3:**
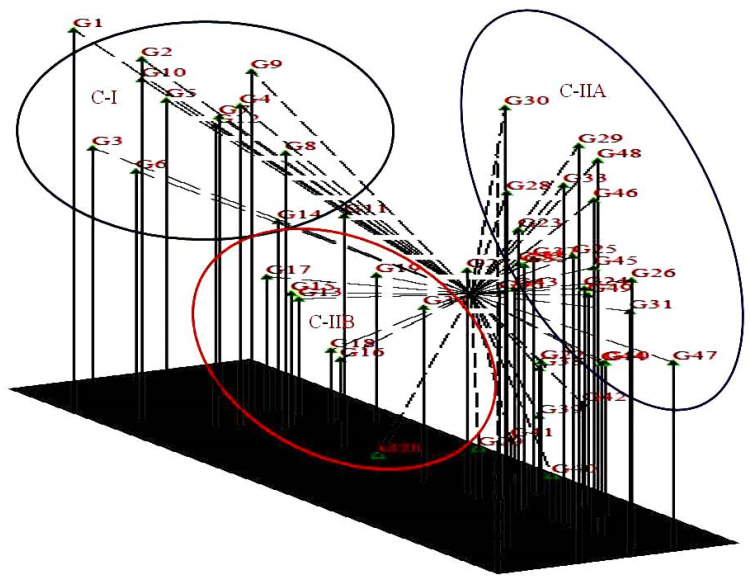
Three-dimensional principal coordinates analysis (PCoA) of 50 apricot genotypes using 46 microsatellite markers.

Cluster I consisted of 10 exotic genotypes divided further into two sub-clusters IA which included 8 genotypes (G1, G3, G4, G5, G6, G7, G8 and G12) and IB, which contained two genotypes (G2 and G10). Cluster II was the largest and contained 39 genotypes that are mainly indigenous to Jammu and Kashmir and form two sub-clusters: cluster IIA and cluster IIB. Cluster IIA is further divided into two sub-clusters and contained 8 genotypes in the first cluster (G9, G11, G13, G14, G34, G35, G36 and G38) and 26 genotypes (G15, G16, G18, G19, G37, G43, G45, G49, G50, G39, G41, G42, G40, G20, G44, G22, G23, G24, G47, G25, G46, G48, G26, G17, G21 and G27). Cluster IIB contained 5 genotypes (G29, G30, G31, G32 and G33) and cluster III contained single genotype G28. The Jaccard’s similarity among the genotypes ranged from 0.508 to 0.867. The highest similarity 0.867 was observed between indigenous accessions G45 and G49, which are accessions from Banihal and Doda regions of Jammu province of J&K and the lowest similarity 0.508 was observed between exotic cultivar Hartley and indigenous accession G31 from Malapora area of Baramullah. A 3D clustering plot revealed that 50 accessions produce three clusters C-I and C-IIA and C-IIB. In addition, the results from 2D PCoA clustering (Figure 2) were consistent with the results of 3DPCoA (Figure 3).

The clustering pattern of apricot accessions from 2D and 3D PCoA plots and the UPGMA clustering graph were highly consistent. The UPGMA clustering tree graph provides abundant information and categorizes the accessions into different groups. The information produced by the 2D PCoA plot, although not sufficient, produced a flat and direct view of the relationship between different accessions as compared to 3D PCoA which provides sufficient information in different layers and directions. The combined result analysis of population structure through genetic similarity and PCoA provides valuable information to understand the genetic structure of the accessions.

### 2.3. Population Structure

**Figure 4:**
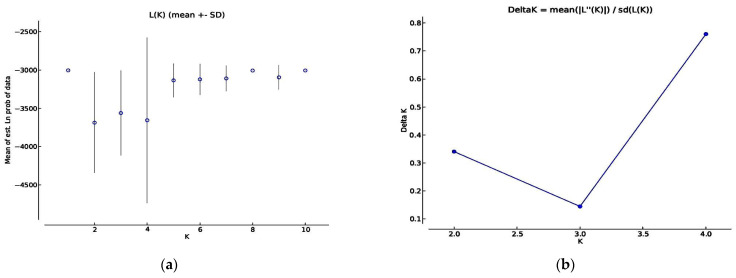
The figures show different methods of calculation of sub-populations (**a**) Non−parametric test showing a probable number of subpopulations using LnP (K) values. (**b**) Delta K showing peak value at K = 4 calculated by Evano et al. (2005) method.

An investigation carried out for population structure utilizing marker data assisted in recognizing four (K = 4) genetically different sub-populations in 50 diverse apricot genotypes. Initially, we were unable to estimate the number of subpopulations as the LnP (K) values decreased from 1 to K = 2 and then increased at K = 3 and then again decreased at K = 4 before subsequently increasing at K = 5 to K = 8 and started again declined at K = 9, and finally at K = 10 increased (Figure 4a). Thus, no comprehensive outcome emerged regarding the probable number of subpopulations using LnP (K) values. Accordingly, to deduce the accurate number of all subpopulations in our population of 50 apricot accessions, the 1K approach developed by Evanno et al. [44] was utilized. The 1K approach calculates the rate of change of the mean probability values (LnP) of all subpopulations. According to this approach, the proportion of change was higher (1830.5) at K = 4 (Figure 4b).

**Figure 5:**
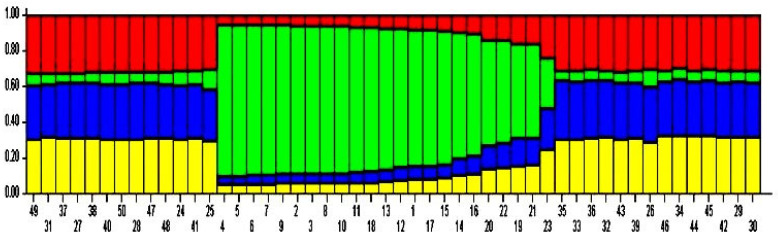
Each column in the figure shows an individual and the X coordinate represents the name of the sample. The length of color represents the proportion of ancestors in the individual genome.

Hence, in our population of 50 apricot accessions, we found 4 subpopulations. Subpopulations 1, 2, 4 contained 36 genotypes that were all indigenous, while as in the 3rd subpopulation, only one indigenous genotype was spotted, the remaining 13 of the genotypes found were exotic. This arrangement pattern was also revealed in the structure graph (Figure 5), depicting the distribution of local (indigenous genotypes) vs. exotic genotypes separately. Further, in the 3rdsubpopulation, all the genotypes had affiliation likelihood more significant than 80%, and hence in this subpopulation, no apricot genotype was displayed as admixture (Table 2). The genotypes in sub-population 1, 2 and 4 have affiliation probability <80%, hence all individuals in these sub-populations were admixtures. The expected heterozygosity was calculated to estimate individuals’ mean distance among and within clusters/subpopulations. The expected heterozygosity, which calculates the likelihood that two randomly selected individuals would be heterozygous at a particular locus, ranged from 1.81 in the third sub-population to 1.77 in the other three sub-populations, with a mean of 1.78. Similarly, population differentiation measurements (Fst) ranged from 0.143 (in the second sub-population) to 0.05 (in the first sub-population), with an average of 0.04 (Table 3).

### 2.4. AMOVA

The purpose of the analysis of molecular variance was to see if there was any genetic variation across populations as well as within populations. According to our results, 95% of the variance was observed within the population, whereas only 5% of the overall genetic diversity was identified between populations (Table 4).

## 3. Discussion

### 3.1. SSR Genotyping and Genetic Diversity Analysis

Microsatellite markers have been successfully employed by several studies to identify molecular genetic variation in apricot genotype collections and populations [2,3,22,45,46,47,48,49,50,51]. In this study, we found most of the amplification bands size range between 90–280 bp, similar size range was observed in cultivated apricot [22,23,24] and peach [52,53]. The large range of allele sizes found revealed a significant amount of genetic distance and diversity among the germplasms examined, which is usually as a result of Russian Botanist Vavilov [54], who considered this zone a rich area of diversity. The diversity indices Na, Ne, Ho, and He were evaluated to assess the degree of genetic variation among wild native apricots and exotic genotypes. The average number of alleles (Na) indicates the richness of alleles in the population and the degree of variability it has [36] and the effective number of alleles (Ne) reflects gene frequency in a population [42]. The observed number of alleles (Na) varied from one to six per locus, and the total number of alleles amplified was 179. Bourguiba et al. [2] reported 609 alleles among 890 worldwide accessions. Among the 46 primers, the highest number of alleles, 6 per locus, was realized with 14 markers, and the highest number of effective alleles (Ne) was identified, two per locus with five markers. In their study, Vilanova et al. [55] reported that the Na ranged from two to seven in apricot accessions. In another study, Zhebentyayeva et al. [24] revealed a higher range of Na 2 to 13 alleles per locus in very diverse germplasm. The lower sample size in our study may have resulted in a lesser number of alleles. The mean Na 3.89 per locus found by us is less than 23.00 found in wild apricot 16.75 [11], Decroocq et al. [3] 6.50 found in landraces [56], 4.00 reported for traditional cultivars [48], 4.27 found in apricot germplasm [7], 4.62 found in common apricot [57], 7.64 reported in endemic apricot cultivars [24] and 15.14 realized in 94 *Prunus* genotypes [26]. The number of alleles was, however, greater than that recorded by Romero et al. [21] in different cultivars (3.1) and almost similar to 3.9 reported by Sanchez-Perez et al. [23]. The average number of effective alleles (Ne) was 1.8821, with a range of 1.1563 to 2. Expected heterozygosity (He) or gene diversity in our investigation varied from 0.13 to 0.50, with an average of 0.45, which was lower than the observed heterozygosity (Ho) of 0.5470. The He range observed by us was narrower in range than 0.4607 to 0.8339 reported by Pedryc et al. [4], 0.37–0.82 by Vilanova et al. [55] and 0.5949–0.8487 by Maghuly et al. [46]. Bourguiba et al. [19] observed that the expected heterozygosity (He) for particular loci differed from 0.04 to 0.82, with a mean value of 0.56 among Tunisian Apricot cultivars. Furthermore, Bourguiba et al. [40] investigated the genetic variability of the apricots grown in Algeria, Morocco and Tunisia and showed expected heterozygosity of 0.593, greater than the average expected 0.45 in this study. Zhang et al. [26] also observed a higher average He of 0.792 in China, Wang et al. [36] observed a He of 0.731 in 150 core samples of Chinese apricot germplasms, Bourguibaet al. [19] revealed that the expected heterozygosity (He) with a mean value of 0.56 among Tunisian Apricot cultivars. The observed heterozygosity ranged from 0 to 1 with a mean value of 0.5226. These values were comparable with 0.51, 0.52, 0.52 reported by Hormaza [22], Raji et al. [24] and Zhebentyayeva et al. [51], respectively, whereas the He value was lesser than 0.58,0.63,0.65,0.68 and 0.72 reported by Ruthner et al. [34], Maghuly et al. [46], Liu et al. [47], Gurcan et al. [58] and Akpinar et al. [59]. The PIC value varied in range from 0.104 to 0.464, with a mean value of 0.320. The average values for PIC in our investigation are less than 0.81 reported by Dehkordi et al. [60]. The Microsatellite sites are the most illuminating ones, those with a greater number of alleles can be utilized directly as DNA fingerprints for apricot cultivar genotype/variety identification. The Shannon information index (I), which estimates diversity, ranged from 0.00 to 0.69 with a mean value of 0.63. Bourgiba et al. [2] found a wider range of I 0.840 to 2.516 with an average value of 2.516. The FST value varied from 0.000 to 0.08, with a mean of 0.022, which was lower than the 0.14, 0.32, 0.38, and 0.5768 reported by Martin et al. [7], Tian-Ming et al. [11], Romero et al. [21], Maghuly et al. [46] and Batnini et al. [50], respectively, in apricot specifying a comparatively low genetic differentiation between genotypes.

### 3.2. Cluster and PCoA Analysis

All genotypes were divided into three main clusters, cluster I, cluster II, and cluster III, with varying degrees of sub-clustering based on the dendrogram. Cluster I comprised ten accessions, the majority of which were exotic genotypes. Cluster I was subdivided into two sub-clusters, IA and IB, which contained eight and two genotypes. Cluster II contained 40 genotypes that are mainly indigenous to Jammu and Kashmir. The grouping of genotypes revealed by the principal coordinate analysis (PCoA), biplot and cluster dendrogram is similar and shows consistency of the results of the grouping of genotypes based on the geographic areas of the sample collection. The first two coordinates of PCoA contributed 68.43% of total genetic variability and the maximum share of this genetic variation is contributed by cluster first (C-I) and cluster IIB (C-IIB). Previously, apricot accessions were arranged using molecular markers according to their geographic origins [12,22,24]. Romero et al. [21] investigated 40 apricot accessions using SSR markers and showed that the accessions were distinguished according to their ecological and geographical origin. According to Zhang et al. [26] and Herrera et al. [56] SSR markers may easily identify natural germplasm or landraces from breeding releases or cultivars. These results also confirm the different genetic nature of exotic and indigenously grown genotypes. These results also show that the members of cluster I had a significant genetic relationship to each other and are genetically distant from other clusters. The similarity coefficient indicated that the highest similarity 0.867 was observed between indigenous accessions 45 (cluster II) and 49 (cluster II), which are from Banihal and Doda areas of Jammu and Kashmir, respectively, and the lowest similarity was observed between exotic cultivar and indigenous accession 2 (cluster I) and 31 (cluster II) (0.508). The highest similarity among the indigenous accessions may be due to the geographical closeness of these genotypes, and the lowest similarity among exotic and indigenous is due to the difference in the genetic makeup of these genotypes.

### 3.3. Population Structure

STRUCTURE analysis of the population is a convincing approach to examine genetic relationships and ancestry of individuals within gene banks [61]. The STRUCTURE revealed four sub-populations and sub-population 1, 2, 4 contained only indigenous accessions and sub-population 3rd contained mostly exotic populations. This arrangement pattern is following the cluster dendrogram, 2D PCoA and 3D PCoA plots depicting the separation of local (indigenous genotypes) vs. exotic genotypes separately. Further, the exotic genotypes in the 3rdsubpopulation show no admixture and each genotype in this sub-population can be considered as genetically pure. The genetically pure nature of these cultivars may be due to the recent inclusion of these genotypes for cultivation in this area. The genotypes in sub-population 1, 2 and 4 were all admixtures. The admixture nature of these genotypes may be due to long periods of gene flow among the genotypes without any geographical barrier. Four genetic subpopulations in our study were also identified as per the accession’s geographical location by [19,49,62]. Zehdi et al. [63] in date palm and Haouane et al. [64] in olive found a similar association between the genetic structure and the geographic origin of the plant material. The expected heterozygosity and population differentiation between and within populations reflected that genetic variations within populations were more substantial than differences among populations and that gene flow among populations was rare [65]. Using the software program STRUCTURE, the allele-frequency deviation between populations (Net nucleotide distance) was calculated by applying point estimation of P. The distance between the two identified subpopulations was found to be 0.2119.

### 3.4. AMOVA

AMOVA revealed a 95% variation within populations and 5.0% of the total molecular variability between populations. Gomez et al. [66] and Vendramin et al. [67], in their findings, also observed an immense amount of genetic deviation occurred within populations of wild apricot (86.3% and 83.6%, respectively). The presence of high variance within the population shows high allelic diversity within populations. This may be due to easy gene flow within individuals of the population than among populations.

## 4. Materials and Methods

### 4.1. DNA Extraction and Amplification

Fresh young tender leaves during preflowering season from each accession were taken in a plastic bag from the field and flash frozen in liquid nitrogen to keep them at −80 °C until DNA extraction. The geo-referenced data, name and exact location of apricot leaf sample collection from the field are shown in Table 5. The germplasm of exotic apricot genotypes were preserved and grown at Central Institute of Temperate Horticulture (CITH), Srinagar. The other indigenous genotypes were grown by farmers in their fields in different districts of Jammu and Kashmir. Furthermore, all the sampled genotypes were phenotypically different showing diverse nature of the experimental study material.

The procedure described by Doyle and Doyle [68] was used to extract genomic DNA. The presence of genomic DNA isolated from 50 genotypes was examined by agarose gel electrophoresis using 1% agarose gel. The purity and amount were examined using a nano-drop spectrophotometer (Thermo Scientific, Waltham, MA, USA). The extracted DNA of each sample was stored at −20 °C after normalization of DNA quantity of each sample to 50 ng/μL for PCR amplification. Forty-six microsatellite markers were used to determine the genetic diversity of apricot samples [69,70,71]. The primers were selected by screening the recent literature related to SSR genetic diversity in apricot and related species, and finally, those primers were selected which have been found polymorphic by evaluating genetic parameters of each primer (Table 6).

PCR reaction was carried out in a 20 μL reaction mixture with 50 ng/µL DNA templates, 10X PCR buffer, 2.5 mM MgCl_2_, 10 mM dNTPs, 1U Taq DNA polymerase, and both primer pairs. A thermal cycler (Takara Thermal Cycler Dice, TD 600, Shiga, Japan) was used for amplification. The PCR amplification steps were executed as initial denaturation for 5 min at 94 °C followed by 35 cycles of 60 s at 94 °C denaturation, 49 to 58 °C for 60 s for optimal annealing temperature for different primers, 90 s at 72 °C for extension and final extension for 10 min at 72 °C followed by cooling at 4 °C. The procedure was performed three consecutive times with the same primers and genotypes to check out the reproducibility. The PCR amplification products and the 100 bp DNA marker were separated on 3% agarose gel with 0.5× TBE buffers using Ethidium bromide (EtBr) as a staining agent on the gel. The banding pattern of the amplified bands was examined under a gel documentation imaging system.

### 4.2. Data Analysis

For all accessions, the composition of alleles and each microsatellite locus were used to calculate the total number of alleles. Indices of molecular characterization were statistically evaluated, including the expected heterozygosity (He), the observed heterozygosity (Ho), the effective number of alleles (Ne), Shannon’s information index (I), the coefficient of gene differentiation (Fst) by applying the POPGENE 1.32 [72,73,74]. In addition to this, based on Jaccard’s similarity coefficient, the Unweighted pair-group method with arithmetic means (UPGMA) hierarchical clustering tree was designed for distinct apricot cultivar groups [75]. STRUCTURE 2.3.4 software was used to analyze ancestral population structure based on Bayesian clustering [76]. STRUCTURE was run ten times, with each run consisting of 100,000-steps followed by 500,000 Markov Chain Monte Carlo (MCMC) iterations, presuming an admixture framework with correlated allelic and several clusters (K) ranging from 1 to 10. The Pritchard et al. [76] criteria and the 1K approach, defined by Evanno et al. [44] and implemented in the STRUCTURE HARVESTER v2.3.4. Websites were used to determine the precise number of populations (K) [77]. CLUMPP v1.1 software [78] was utilized using optimistic algorithms, 10,000 random input orders, and 10,000 repeats to estimate the mean pairwise similarity of runs and produce optimum alignment of independent runs. To graphically display the results, the output files of CLUMPP were used as input files for DISTRUCT v1.1 software, the output of CLUMPP was immediately fed into DISTRUCT v1.1 [79]. The probability membership of each accession was ascertained, they were allocated to the appropriate cluster if their affiliation was higher than 80%; otherwise, they were labeled admixture. For estimation of genetic differentiation among and within populations, AMOVA analysis was done in software GenAlEx v6.503 [80].

## 5. Conclusions

In conclusion, our investigation has dispensed a broader context on genetic variability and core structure among apricot accessions in Jammu and Kashmir. The results revealed that the SSR marker studies could easily decrypt the genetic variability present within the germplasm. This was the first kind of study carried out in this area to distinguish exotic genotypes from indigenous genotypes via molecular markers and showed a high level of polymorphism. Genetic variability between exotic and indigenous genotypes can provide an excellent opportunity for new cultivar development through hybridization and advanced genetic tools such as molecular markers. These diversity analysis tools could be utilized for the establishment and collection of gene banks and core collections by reducing redundancy of germplasm, selection of parents for any breeding program and genome-wide association studies for mapping of different traits.

## Figures and Tables

**Table 1:** Genetic parameters of 46 SSR markers evaluated on 50 *Prunus armeniaca* L. accessions.

Marker	No. of Alleles	PIC Value	Obs Hom	Obs Het	Exp Hom	Exp Het	Ave Het	Ne	I	Fst
RPPG1-017	6	0.374	0.8333	0.1667	0.7193	0.2807	0.2778	1.3846	0.4506	0.05
RPPG1-026	6	0.350	0.5833	0.4167	0.5088	0.4912	0.4861	1.9459	0.6792	0.02
RPPG1-032	5	0.362	0.5417	0.4583	0.5026	0.4974	0.4922	1.9692	0.6853	0.04
RPPG1-037	3	0.283	0.5208	0.4792	0.5055	0.4945	0.4894	1.9584	0.6825	0.07
RPPG1-041	2	0.196	0.7083	0.2917	0.5509	0.4491	0.4444	1.8	0.6365	0.02
RPPG2-011	1	0.104	0.4167	0.5833	0.5825	0.4175	0.4132	1.7041	0.6036	0
RPPG2-022	1	0.104	0.8542	0.1458	0.8634	0.1366	0.1352	1.1563	0.2611	0.08
RPPG3-026	2	0.186	0.2917	0.7083	0.4982	0.5018	0.4965	1.9862	0.6897	0.01
RPPG4-059	4	0.350	0.6458	0.3542	0.5125	0.4875	0.4824	1.9321	0.6755	0.01
RPPG4-067	2	0.203	0.9375	0.0625	0.5318	0.4682	0.4633	1.8633	0.656	0.04
RPPG4-077	3	0.284	0.2917	0.7083	0.5377	0.4623	0.4575	1.8432	0.65	0
RPPG4-084	2	0.194	0.7083	0.2917	0.5263	0.4737	0.4688	1.8824	0.6616	0.06
RPPG4-091	6	0.462	0.2917	0.7083	0.4982	0.5018	0.4965	1.9862	0.6897	0
RPPG5-018	4	0.326	0.4167	0.5833	0.5825	0.4175	0.4132	1.7041	0.6036	0
RPPG5-022	3	0.284	0.4792	0.5208	0.495	0.505	0.4998	1.9991	0.6929	0.05
RPPG5-023	2	0.188	0.5208	0.4792	0.5055	0.4945	0.4894	1.9584	0.6825	0
RPPG5-025	1	0.105	0.7292	0.2708	0.5055	0.4945	0.4894	1.9584	0.6825	0.03
RPPG5-030	4	0.354	0.000	1.000	0.4947	0.5053	0.500	2.000	0.6931	0.01
RPPG6-009	4	0.351	0.5	0.5	0.5167	0.4833	0.4783	1.9168	0.6713	0.02
RPPG6-032	4	0.354	0.5625	0.4375	0.495	0.505	0.4998	1.9991	0.6929	0
RPPG6-033	2	0.190	0.7083	0.2917	0.4947	0.5053	0.500	2.000	0.6931	0.05
RPPG7-015	4	0.353	0.2708	0.7292	0.5055	0.4945	0.4894	1.9584	0.6825	0.02
RPPG7-026	4	0.354	0.5417	0.4583	0.643	0.357	0.3533	1.5463	0.5383	0.04
RPPG7-032	3	0.279	0.875	0.125	0.4956	0.5044	0.4991	1.9965	0.6923	0
RPPG8-007	1	0.107	0.875	0.125	0.5026	0.4974	0.4922	1.9692	0.6853	0.03
RPPG8-028	3	0.260	0.1875	0.8125	0.5125	0.4875	0.4824	1.9321	0.6755	0.01
Aprigms18	5	0.412	0.3542	0.6458	0.495	0.505	0.4998	1.9991	0.6929	0
UDP98-405	6	0.447	0.5833	0.4167	0.5825	0.4175	0.4132	1.7041	0.6036	0
UDP98-406	6	0.462	0.375	0.625	0.5026	0.4974	0.4922	1.9692	0.6853	0.02
UDP98-409	4	0.337	0.125	0.875	0.5026	0.4974	0.4922	1.9692	0.6853	0.05
UDP98-411	6	0.464	0.0833	0.9167	0.4982	0.5018	0.4965	1.9862	0.6897	0.05
Pchgms4	6	0.463	0.8333	0.1667	0.7789	0.2211	0.2188	1.2800	0.3768	0.01
Pchgms5	5	0.414	1.000	0.000	0.8114	0.1886	0.1866	1.2295	0.3341	0.06
Bppct007	6	0.464	0.5833	0.4167	0.5509	0.4491	0.4444	1.800	0.6365	0.01
Bppct025	2	0.193	0.375	0.625	0.5658	0.4342	0.4297	1.7534	0.6211	0
Bppct030	6	0.462	0.3542	0.6458	0.5441	0.4559	0.4512	1.8221	0.6435	0.05
PacA10	6	0.462	0.000	1.000	0.4947	0.5053	0.5000	2.000	0.6931	0
PacA18	6	0.464	0.2083	0.7917	0.4956	0.5044	0.4991	1.9965	0.6923	0.04
PacA33	6	0.463	0.2292	0.7708	0.5213	0.4787	0.4737	1.9002	0.6667	0.01
PacA22	6	0.460	0.125	0.875	0.4947	0.5053	0.500	2.000	0.6931	0
PacA26	6	0.462	0.375	0.625	0.4947	0.5053	0.500	2.000	0.6931	0.02
PacA35	4	0.35	0.500	0.500	0.6211	0.3789	0.375	1.600	0.5623	0.01
PacC3	2	0.20	0.4167	0.5833	0.5088	0.4912	0.4861	1.9459	0.6792	0.02
PacC25	3	0.27	0.4792	0.5208	0.5739	0.4261	0.4217	1.7291	0.6126	0.02
PacA58	2	0.190	0.3125	0.6875	0.495	0.505	0.4998	1.9991	0.6929	0
PdavW3	4	0.352	0.3542	0.6458	0.5441	0.4559	0.4512	1.8221	0.6435	0.02
Mean	3.89	0.320	0.4774	0.5226	0.5470	0.4530	0.4483	1.8221	0.6371	0.0228

Legend: (Exp Ho) Expected homozygosity, (Exp He) heterozygosity, (Ob He) observed heterozygosity, (Ob Ho) homozygosity, (Ave Het) Average Heterozygosity, Ne = Effective number of alleles, I = Shannon’s information index, Fst = Genetic differentiation.

**Table 2:** Distribution of individuals to sub-populations (K) on the basis of genetic ancestry.

Code	Genotype	K1	K2	K3	K4	Sub-Population
1	G1	0.049	0.045	0.861	0.045	3
2	G2	0.029	0.025	0.923	0.024	3
3	G3	0.028	0.025	0.92	0.027	3
4	G4	0.022	0.023	0.935	0.021	3
5	G5	0.021	0.02	0.937	0.022	3
6	G6	0.024	0.025	0.928	0.024	3
7	G7	0.024	0.024	0.928	0.024	3
8	G8	0.032	0.028	0.915	0.026	3
9	G9	0.035	0.027	0.911	0.027	3
10	G10	0.029	0.026	0.918	0.027	3
11	G11	0.033	0.03	0.903	0.034	3
12	G12	0.084	0.057	0.807	0.051	3
13	G13	0.075	0.047	0.833	0.046	3
14	G14	0.101	0.077	0.751	0.071	Admixture of 1,2,3,4
15	G15	0.101	0.069	0.76	0.07	Admixture of 1,2,3,4
16	G16	0.116	0.081	0.719	0.084	Admixture of 1,2,3,4
17	G17	0.094	0.062	0.783	0.061	Admixture of 1,2,3,4
18	G18	0.036	0.034	0.897	0.033	3
19	G19	0.178	0.146	0.53	0.146	Admixture of 1,2,3,4
20	G20	0.16	0.123	0.595	0.122	Admixture of 1,2,3,4
21	G21	0.168	0.156	0.516	0.16	Admixture of 1,2,3,4
22	G22	0.15	0.136	0.579	0.134	Admixture of 1,2,3,4
23	G23	0.248	0.239	0.287	0.227	Admixture of 1,2,3,4
24	G24	0.3	0.328	0.047	0.325	Admixture of 1,2,3,4
25	G25	0.296	0.317	0.078	0.309	Admixture of 1,2,3,4
26	G26	0.312	0.326	0.057	0.306	Admixture of 1,2,3,4
27	G27	0.323	0.318	0.024	0.335	Admixture of 1,2,3,4
28	G28	0.329	0.311	0.028	0.333	Admixture of 1,2,3,4
29	G29	0.329	0.313	0.027	0.331	Admixture of 1,2,3,4
30	G30	0.298	0.339	0.03	0.333	Admixture of 1,2,3,4
31	G31	0.318	0.321	0.024	0.337	Admixture of 1,2,3,4
32	G32	0.324	0.319	0.02	0.337	Admixture of 1,2,3,4
33	G33	0.315	0.332	0.03	0.322	Admixture of 1,2,3,4
34	G34	0.307	0.327	0.028	0.338	Admixture of 1,2,3,4
35	G35	0.315	0.335	0.022	0.328	Admixture of 1,2,3,4
36	G36	0.311	0.321	0.025	0.342	Admixture of 1,2,3,4
37	G37	0.314	0.317	0.024	0.345	Admixture of 1,2,3,4
38	G38	0.308	0.337	0.025	0.329	Admixture of 1,2,3,4
39	G39	0.323	0.32	0.032	0.325	Admixture of 1,2,3,4
40	G40	0.308	0.337	0.033	0.322	Admixture of 1,2,3,4
41	G41	0.316	0.33	0.038	0.316	Admixture of 1,2,3,4
42	G42	0.319	0.323	0.029	0.329	Admixture of 1,2,3,4
43	G43	0.318	0.328	0.025	0.329	Admixture of 1,2,3,4
44	G44	0.33	0.32	0.029	0.322	Admixture of 1,2,3,4
45	G45	0.299	0.316	0.029	0.356	Admixture of 1,2,3,4
46	G46	0.312	0.327	0.033	0.328	Admixture of 1,2,3,4
47	G47	0.314	0.326	0.026	0.334	Admixture of 1,2,3,4
48	G48	0.305	0.34	0.035	0.32	Admixture of 1,2,3,4
49	G49	0.314	0.328	0.031	0.327	Admixture of 1,2,3,4
50	G50	0.296	0.327	0.028	0.349	Admixture of 1,2,3,4

**Table 3:** Heterozygosity and Fst value of four sub-populations of the apricot.

S. No	Sub-Population	Exp Het	Fst
01	1	1.77	0.005
02	2	1.81	0.143
03	3	1.77	0.006
04	4	1.77	0.006
Average		1.78	0.04

**Table 4:** Summary AMOVA table.

Source	df	SS	MS	Est. Var.	%
Among populations	1	32.385	32.385	0.557	5%
Within populations	98	973.665	9.935	9.935	95%
Total	99	1006.050		10.492	100%

**Table 5:** Geographical coordinates and location of Apricot accessions evaluated in this study.

S.NO	Genotype Name	Code	Location	District	Latitude	Longitude	Origin
1	‘Harcot’	G1	CITH	Budgam	33.9749° N	74.7895° E	Exotic
2	‘Hartlay’	G2	CITH	Budgam	33.9741° N	74.7889° E	Exotic
3	‘Irani’	G3	CITH	Budgam	33.9739° N	74.7884°E	Exotic
4	‘Communis-Holi’	G4	CITH	Budgam	33.9725° N	74.7882° E	Exotic
5	‘Tilton’	G5	CITH	Budgam	33.9719° N	74.7875° E	Exotic
6	‘Rival’	G6	CITH	Budgam	33.9716° N	74.7872° E	Exotic
7	‘Tokpopanimu’	G7	CITH	Budgam	33.9711° N	74.7863° E	Exotic
8	‘Fair medister’	G8	CITH	Budgam	33.9706° N	74.7858° E	Exotic
9	‘Viva Gold’	G9	CITH	Budgam	33.9701° N	74.7850° E	Exotic
10	‘Cummins’	G10	CITH	Budgam	33.9721° N	74.7877° E	Exotic
11	‘Turkey’	G11	CITH	Budgam	33.9714° N	74.7867° E	Exotic
12	‘New-Castle’	G12	CITH	Budgam	33.9731° N	74.7881°E	Exotic
13	‘Chinese Apricot’	G13	CITH	Budgam	33.9752° N	74.7497° E	Exotic
14	Unknown	G14	Hardas	Ladakh	34.6061° N	76.0981° E	Indigenous
15	Unknown	G15	Ushkara	Baramulla	34.2504° N	74.3788° E	Indigenous
16	Unknown	G16	Chardari	Baramulla	34.1852° N	74.3634° E	Indigenous
17	Unknown	G17	Kantibag	Baramulla	34.2406° N	74.3674° E	Indigenous
18	Unknown	G18	Uri	Baramulla	34.0831° N	74.0543° E	Indigenous
19	Unknown	G19	Rangwar	Baramulla	34.2343° N	74.3676° E	Indigenous
20	Unknown	G20	Beerwah	Budgam	34.0128° N	74.5956° E	Indigenous
21	Unknown	G21	Katiyawali	Baramulla	34.1754° N	74.3531° E	Indigenous
22	unknown	G22	Gatha Baderwah	Doda	32.9973° N	75.7007° E	Indigenous
23	unknown	G23	Khanpora	Baramulla	34.2086° N	74.3275° E	Indigenous
24	unknown	G24	Brazllo	Kulgam	33.6467° N	75.0589° E	Indigenous
25	unknown	G25	Shiva	Baramulla	34.3521° N	74.4748° E	Indigenous
26	unknown	G26	Dogar	Baramulla	33.1829° N	74.3619° E	Indigenous
27	unknown	G27	Narapora	Shopian	34.7611° N	74.8019° E	Indigenous
28	unknown	G28	Buniyar	Baramulla	34.1009° N	74.2004° E	Indigenous
29	unknown	G29	Gozahama	Ganderbal	34.1934° N	74.6755° E	Indigenous
30	unknown	G30	Kokarnag	Anantnag	33.6801° N	75.3895° E	Indigenous
31	unknown	G31	Malpora	Baramulla	34.3528° N	74.4732° E	Indigenous
32	unknown	G32	Dangerpora	Pulwama	33.8756° N	74.9793° E	Indigenous
33	unknown	G33	Sopore	Baramulla	34.2604° N	74.4681° E	Indigenous
34	unknown	G34	Duroo	Baramulla	34.3516° N	74.4633° E	Indigenous
35	unknown	G35	Pazelpora	Baramulla	34.3587° N	74.4831° E	Indigenous
36	unknown	G36	Kanispora	Baramulla	34.2184° N	74.3998° E	Indigenous
37	unknown	G37	Darpora	Baramulla	34.3570° N	74.4323° E	Indigenous
38	unknown	G38	Goripora	Baramulla	34.3465° N	74.4212° E	Indigenous
39	unknown	G39	Mundji	Baramulla	34.3607° N	74.4738° E	Indigenous
40	unknown	G40	Handwara	Kupwara	34.4043° N	74.2831° E	Indigenous
41	unknown	G41	Brath Kalan	Baramulla	34.3446° N	74.4065° E	Indigenous
42	unknown	G42	Wadura	Baramulla	34.3528° N	74.4018° E	Indigenous
43	unknown	G43	Badwenchak	Qazigund	33.5927° N	75.1658° E	Indigenous
44	unknown	G44	Sheeri	Baramulla	34.1107° N	74.1837° E	Indigenous
45	unknown	G45	Krawah	Banihal	33.2518° N	75.1048° E	Indigenous
46	unknown	G46	Chadoora	Budgam	33.9453° N	75.7967° E	Indigenous
47	unknown	G47	Kralpora	Budgam	34.4997° N	74.1177° E	Indigenous
48	unknown	G48	Bhangra	Doda	32.9831° N	75.7116° E	Indigenous
49	unknown	G49	KapraBaderwah	Doda	32.9833° N	75.7112° E	Indigenous
50	unknown	G50	Rawalpora	Srinagar	34.0042° N	74.4676° E	Indigenous

**Table 6:** List of evaluated SSR markers screened with their primer sequence and allele size range calculated in apricot genotypes studied.

SSR Marker	Primer Sequence 5′→3′	Reference	Size Range (bp)
RPPG1-017	F:GCTCATCAAAACTCTCAACCA R:CCCTTTCTTCAATCCCATC	Dettori et al., 2015	90–220
RPPG1-026	F:CTTCTGGCACTCTTCCATTT R:GTTCCCAAGTTTTCCTCTCA	Dettori et al., 2015	90–220
RPPG1-032	F:ATGGCAGAGAGCACAACAA R:TTGAGAGGTAACAGCGAGAA	Dettori et al., 2015	90–250
RPPG1-037	F:GTCTCTGATCCAAGCCAACT R:ACGCTGCCATTGTTTCTATT	Dettori et al., 2015	100–250
RPPG1-041	F:TGTTGTAATGGATGGTGTCTTC R:CTTGGTCTTGGTTTCATTCA	Dettori et al., 2015	120–220
RPPG2-011	F:TTTACAGGTGCCTCAACAAA R:GTACAGCCGATGGAGAGAAA	Dettori et al., 2015	180
RPPG2-022	F:CTGCTGCGTCTGATGATG R:ACAGGACAGGACCACTTTCT	Dettori et al., 2015	200
RPPG3-026	F:AGAACGCTATTCCCCTGTAA R:TCATCCTCTCCAAATGTCAA	Dettori et al., 2015	90–200
RPPG4-059	F:GACGGCTGTTTATTTGCATT R:TGCATTTGTGATCTCGTTTC	Dettoriet al., 2015	100–180
RPPG4-067	F:AGAAGGGAGGGTGAGAGAAG R:CACGAAGGAAGAAACGAAGT	Dettori et al., 2015	100–210
RPPG4-077	F:CCTCGTCTTCAGTCTTTTCTG R:CTGTCCCTTCTGTGTTCCTAA	Dettori et al., 2015	90–150
RPPG4-084	F:TCCTCAAAAGTTACCCCAAG R:CTTGCTGTGGAAGAAGAACC	Dettori et al., 2015	120–200
RPPG4-091	F:GGAGGGTAGAGAACAGAGCA R:CGGAAGATGTGATTGTGAGA	Dettori et al., 2015	90–220
RPPG5-018	F:GCATGAAATTGACCCATACA R:TAATTGCTTTGGGGAGGAC	Dettori et al., 2015	90–200
RPPG5-022	F:CTTGTGAACTGGCATCTGTC R:AGTTGTATGGGCATGTTGTG	Dettori et al., 2015	90–180
RPPG5-023	F:TTGTTTGCACTAGGCTTTGA R:TTCTTCTTGCATGTCCTTGA	Dettori et al., 2015	90–150
RPPG5-025	F:GTGTCTCCTCCTCAAAGCAA R:TACGGCAACCAAGAACATC	Dettori et al., 2015	120
RPPG5-030	F:AAGGCAAGGAATTGGGTAGT R:TGGTTTGTCGTAAGAGTCCA	Dettori et al., 2015	90–280
RPPG6-009	F:GGGCTTGGCTGATAAAATAA R:TGGTAAAATAGAAGAGCGAGAAG	Dettori et al., 2015	100–120
RPPG6-032	F:TCCTATGGCAAAAACAAAATC R:TGAAGAGATGGAGTGGAAGAG	Dettori et al., 2015	90–150
RPPG6-033	F:CATTATCAAACCACGACCAA R:AAAGCTCAACAGCGACTTCT	Dettori et al., 2015	100–200
RPPG7-015	F:TCTTGGTGGTGGTGAAGTAA R:GAGAGATGGAGGAGGCTGA	Dettori et al., 2015	90–180
RPPG7-026	F:TTTGGTGAGTGGGCTCTATT R:CTATCGTTCGCTGGTCTTCT	Dettori et al., 2015	90–180
RPPG7-032	F:AAGGGAGGAGGATTGTGAA R:TGGTAGACGGGTAGATGTTG	Dettori et al., 2015	90–180
RPPG8-007	F:ACCACCACCTCTTCCAATC R:ACCTCAAAGTGTCCCAGAAA	Dettori et al., 2015	150
RPPG8-028	F:AAGGAGCCGACATCAGAAC R:TGACCAGAAGCCAAATACATC	Dettori et al., 2015	120–180
Aprigms18	F:TCTGAGTTCAGTGGGTAGCA R:ACAGAATGTGCGTTGCTTTA	Liu et al., 2015	90–200
UDP98-405	F:ACGTCATGAACTGACACCCA R:GAGTCTTTGCTCTGCCATCC	Liu et al., 2015	90–120
UDP98-406	F:TCGGAAACTGGTAGTATGAACAGA R:ATGGGTCGTATGCACAGTCA	Liu et al., 2015	90–120
UDP98-409	F:GCTGATGGGTTTTATGGTTTTC R:CGGACTCTTATCCTCTATCAACA	Liu et al., 2015	90–150
UDP98-411	F:AAGCCATCCACTCAGCACTC R:CCAAAAACCAAAACCAAAGG	Liu et al., 2015	90–180
Pchgms4	F:ATCTTCACAACCCTAATGTC R:GTTGAGGCAAAAGACTTCAAT	Liu et al., 2015	90–280
Pchgms5	F:CGCCCATGACAAACTTA R:GTCAAGAGGTACACCAG	Liu et al., 2015	150–280
Bppct007	F:TCATTGCTCGTCATCAGC R:CAGATTTCTGAAGTTAGCGGTA	Liu et al., 2015	150–420
Bppct025	F:TCCTGCGTAGAAGAAGGTAGC R:CGACATAAAGTCCAAATGGC	Liu et al., 2015	90–150
Bppct030	F:AATTGTACTTGCCAATGCTATGA R:CTGCCTTCTGCTCACACC	Liu et al., 2015	90–180
PacA10	F:TGAGCATAATTGGGGCAG R:GCCAGAGAAGCCATTTCAGT	Lambert et al., 2004	120–250
PacA18	F:TCCAAACCTACCGTTTCTCAT R:CAACAGCACAAACAGAACCAC	Lambert et al., 2004	180–250
PacA33	F:TCAGTCTCATCCTGCATACG R:CATGTGGCTCAAGGATCAAA	Lambert et al., 2004	90–250
PacA22	F:AACCAGTTGCCTCTAGATTTTG R:AGCTGAAAGTCAATTCAGAGTAGTT	Lambert et al., 2004	100–180
PacA26	F:CCAATCATGAAAATCATAAAAGCAA R:TGGGATGTCCTATTGTTTTCA	Lambert et al., 2004	100–200
PacA35	F:ATTGCGATTTCGGTCTGTT R:CCATCCCAAATTGCTTACTT	Lambert et al., 2004	120–180
PacC3	F:TGACTTGATCAGACTCGACA R:TTGCATTTGCATTTACAATAGA	Lambert et al., 2004	90–200
PacC25	F:GTGTTTTGACAAGAAATGAATTG R:TCCATTCGCAGTAAAATTAAAC	Lambert et al., 2004	100–200
PacA58	F:GACATTGCGATTTCGGTC R:TCCATCCCAAATTGCTTACT	Lambert et al., 2004	100–180
PdavW3	F:GAGGGCTGGATCATGACG R:AACCCAGTGGCACAATCGTA	Lambert et al., 2004	90–200

## Data Availability

Data are contained within the article.
